# Cellular Cullin RING Ubiquitin Ligases: Druggable Host Dependency Factors of Cytomegaloviruses

**DOI:** 10.3390/ijms20071636

**Published:** 2019-04-02

**Authors:** Tanja Becker, Vu Thuy Khanh Le-Trilling, Mirko Trilling

**Affiliations:** Institute for Virology, University Hospital Essen, University Duisburg-Essen, 45147 Essen, Germany; Tanja.Becker@uk-essen.de (T.B.); Khanh.Le@rlk.uk-essen.de (V.T.K.L.-T.)

**Keywords:** human cytomegalovirus, MLN4924, DDB1, Cullin RING ubiquitin ligases, antiviral drugs

## Abstract

Human cytomegalovirus (HCMV) is a ubiquitous betaherpesvirus that frequently causes morbidity and mortality in individuals with insufficient immunity, such as transplant recipients, AIDS patients, and congenitally infected newborns. Several antiviral drugs are approved to treat HCMV infections. However, resistant HCMV mutants can arise in patients receiving long-term therapy. Additionally, side effects and the risk to cause birth defects limit the use of currently approved antivirals against HCMV. Therefore, the identification of new drug targets is of clinical relevance. Recent work identified DNA-damage binding protein 1 (DDB1) and the family of the cellular cullin (Cul) RING ubiquitin (Ub) ligases (CRLs) as host-derived factors that are relevant for the replication of human and mouse cytomegaloviruses. The first-in-class CRL inhibitory compound Pevonedistat (also called MLN4924) is currently under investigation as an anti-tumor drug in several clinical trials. Cytomegaloviruses exploit CRLs to regulate the abundance of viral proteins, and to induce the proteasomal degradation of host restriction factors involved in innate and intrinsic immunity. Accordingly, pharmacological blockade of CRL activity diminishes viral replication in cell culture. In this review, we summarize the current knowledge concerning the relevance of DDB1 and CRLs during cytomegalovirus replication and discuss chances and drawbacks of CRL inhibitory drugs as potential antiviral treatment against HCMV.

## 1. The Human Cytomegalovirus

Human cytomegalovirus (HCMV, also called human herpesvirus 5 [HHV-5], taxonomy ID: 10359) is the prototypic member of the betaherpesvirus subfamily. The majority of the global human population is latently infected with HCMV. The seroprevalence of a given group depends on the age, gender, and socioeconomic status of its members. While in developing countries almost the entire adult population is infected with HCMV, the incidence is lower in developed countries like in Germany [1], where a seroprevalence of 56.7% was recently reported [2]. In the large majority of the cases, the primary HCMV infection of healthy adults causes subclinical or mild symptoms, since the immune system controls the viral replication. Although the concerted activity of the intrinsic, innate, and adaptive arms of the immune system usually suppresses HCMV below the threshold for clinical detection, clearance is never achieved. In contrast, HCMV inevitably establishes a state of life-long latency, from which it can reactivate under immunocompromising or stressful circumstances, causing recurrent episodes of virus shedding. With only few notable exceptions in apparently healthy adults [3], HCMV infection typically becomes clinically relevant under conditions of impaired, senescent, or immature immunity. For instance, HCMV is a highly relevant opportunistic pathogen in human immunodeficiency virus (HIV)-infected patients, in particular during the phase of acquired immunodeficiency syndrome (AIDS). Especially in regions where the highly active antiretroviral therapy (HAART) is either not broadly available or patients hold off from medical help, e.g., due to social stigmatization, HCMV co-infections can cause retinitis, colitis, esophagitis, pneumonitis, neurological disorders, and several other manifestations [4]. Even in HAART-treated HIV patients, HCMV co-infections are associated with enhanced HIV progression and early-onset of age-related diseases [5].

Another cohort of patients particularly vulnerable to HCMV infections and disease are transplant recipients, who rely on immunosuppressive therapies to avoid transplant rejection and/or graft-versus-host disease (GvHD). Despite the availability of reliable molecular diagnostics and several Food and Drug Administration (FDA)-approved antiviral drugs (see below), the HCMV infection of the donor and/or recipient, as indicated by their serostatus, still significantly influences the mortality and morbidity of transplant recipients [6,7]. The most dangerous constellation is the presence of HCMV infection in the absence of HCMV-specific immune cells, e.g., D+/R- in solid organ transplantations and D-/R+ in bone marrow transplantations.

Primary HCMV infections, reactivations, and reinfections during pregnancy can lead to HCMV transmission in utero from the pregnant mother via the placenta to the developing fetus. Therefore, HCMV is classified as one of the so-called TORCH infections (toxoplasmosis, rubella, cytomegalovirus, herpes simplex, and other organisms including syphilis, parvovirus, Zika, and Varicella zoster), a group of congenitally acquired infections causing significant morbidity and mortality in neonates. Infection of the fetus during pregnancy can lead to mental retardation, microcephaly, sensorineural hearing loss, and other sequelae. Although HCMV is the most frequent cause of pathogen-induced congenital defects and certain hygiene precautions reduce the likelihood of HCMV acquisition, the awareness among women of child-bearing age is alarmingly low [8,9].

Given this clinical relevance of HCMV, the National Academy of Medicine assigned top priority to an HCMV vaccine already two decades ago. However, until now and despite several significant attempts, no HCMV vaccine is currently available [10,11,12].

HCMV is an enveloped virus with an icosahedral capsid harboring a ~235 kbp double-stranded DNA (dsDNA) genome. The genome is composed of a unique long (UL) and a unique short (US) region. The US region of all known HCMVs and the UL region of fibroblast-adapted strains (e.g., AD169varS) are surrounded by inverted repeats (R). According to their association, the repeats of the UL region are called repeat long (RL), and repeats surrounding the US region are called repeat short (RS). The HCMV genes are named according to their position in the respective genome region [13]. For example, *UL145* is the one hundred and forty-fifth gene in the UL region. The genes of the related mouse cytomegalovirus (MCMV), which serves as established model to explore the pathology and the immune recognition of CMVs (see e.g., [14]), are also named according to their genome position (*m01*-*m170*). If a homologous HCMV gene exists, the ‘m’ is capitalized (e.g., *M27*). However, recent data revealed a very complex coding capacity of HCMV comprising e.g., internal and overlapping open reading frames (ORFs), splicing sites, and expression from both strands, resulting in several hundred different transcripts and translation products [15,16,17,18]. This challenges the gene nomenclature based on early genome annotations. Although not all transcripts give rise to stable polypeptides, many gene products fulfill important functions beyond the genome replication or as structural component of the virion. Numerous viral proteins modulate the host organism to establish an appropriate environment for viral replication, e.g., by contributing to viral immune evasion.

### Currently Available Antiviral Drugs Against Cytomegaloviruses

HCMV-associated diseases can be treated by antiviral drugs like (Val-) Ganciclovir, Letermovir, Foscarnet, and Cidofovir. Some of these FDA-approved drugs are widely prescribed to transplant recipients as well as HIV patients and have undoubtedly prevented countless fatalities and helped numerous patients (see e.g., [19]). However, they have limitations due to their toxicity and teratogenicity. Additionally, long-term usage can lead to the development of antiviral drug resistance. The nucleoside analogues Ganciclovir (GCV) and its oral prodrug Valganciclovir exhibit side-effects including hematologic toxicity manifested as leukopenia, neutropenia, anemia, or thrombocytopenia [20]. The pyrophosphate analogue Foscarnet and the nucleotide analogue Cidofovir are associated with nephrotoxicity [21]. Moreover, based on animal experiments, these drugs are suspected to be teratogenic and/or embryotoxic, arguing against their use during pregnancy (see e.g., [22]). Upon GCV treatment, resistance-associated mutations in the genes encoding the viral kinase (*UL97*) and/or the viral DNA polymerase (*UL54*) arise. For Foscarnet and Cidofovir, mutations conferring antiviral resistance map to the viral DNA polymerase [23,24]. A lipid-conjugated form of Cidofovir was developed under the name Brincidofovir (CMX001). Despite its reduced nephrotoxicity, Brincidofovir failed to show superior clinical outcomes in preventing clinically significant CMV infections in hematopoietic stem cell transplantation HSCT patients when compared to a placebo treatment. Furthermore, the overall mortality was even higher in patients receiving Brincidofovir as compared to the placebo group [25]. The FDA- and European Medicines Agency (EMA)-approved anti-sense oligonucleotide Fomivirsen (Vitravene), which was used to treat HCMV retinitis, was withdrawn after the introduction of anti-HIV HAART due of the reduced number of HCMV retinitis cases [26]. The pUL97-specific kinase inhibitor Maribavir stimulated great expectations in the field [27]. Unfortunately, it did not reach the primary endpoint to reduce HCMV-associated diseases in a placebo-controlled, randomized, double-blind, multicenter phase three study in recipients of stem-cell transplantation [28]. There may be a beneficial effect of Maribavir treatment for HCMV infections refractory or resistant to (Val-) Ganciclovir, Foscarnet, and Cidofovir [29]. However, the benefit of Maribavir needs to be confirmed in future studies comprising a Non-Maribavir comparator arm. Based on an excellent clinical performance, Letermovir, a very promising new HCMV DNA terminase inhibitor, was recently approved [30]. The DNA terminase complex cleaves viral DNA concatemers into DNA monomers. However, HCMV can acquire Letermovir resistance in vitro [31] and in patients [32]. The resistance was associated with mutations in *UL56* and to lesser extend in *UL89* or *UL51* [33,34]. Due to these limitations, we are convinced that additional treatment options and drug targets are urgently needed.

## 2. Interferons

Interferons (IFN) are a family of very potent antiviral cytokines [35]. Upon infection, pathogen-associated molecular patterns are recognized by the cognate receptors. Via a complex network of receptor-associated adaptor proteins, kinases, and transcription factors, the expression of IFN genes is induced. Upon binding of secreted IFNs to their cell surface receptors, a signaling cascade based on janus kinases (Jak), as well as signal transducer and activator of transcription (STAT) proteins is engaged [36]. The Jak-dependent tyrosine phosphorylation activates STATs. The STAT proteins homo- or heterodimerize, translocate into the nucleus, bind to specific DNA elements, and induce the transcription of adjacent IFN-stimulated genes (ISGs). Based on the homology and the receptor usage, IFNs are subdivided into type I IFNs (IFN-I), type II IFNs (IFN-II), and type III IFNs (IFN-III) comprising IFNα/β, IFNγ, and IFNλ, respectively. IFN-I and IFN-III have their own specific receptor complexes but seem to activate similar signaling cascades, which mainly rely on tyrosine kinase 2 (Tyk2), Jak1, STAT1, STAT2, and IFN-regulatory factor (IRF) 9 [37]. Conversely, the canonical IFNγ signaling is based on Jak1 and Jak2, which induce the phosphorylation and activation of STAT1 homodimers. However, IFNγ also stimulates a non-canonical signal transactivation [38] like the activation of the IFN-stimulated gene factor 3 (ISGF3) composed of STAT1, STAT2, and IRF9 [39,40,41,42,43,44]. Recently, it has become evident that IFNγ also represses the expression of numerous genes termed IFN-repressed genes (IRepGs) [43,45,46]. In combination, the IFN-induced alterations of ISGs and IRepGs elicit antiviral activity [45,46].

Based on their potent antiviral activity, IFNs have been approved and are successfully used as antiviral drugs, e.g., against hepatitis viruses. However, with the development of effective direct acting antiviral (DAA) drugs, e.g., against hepatitis C virus (HCV), the use of IFNs is declining.

In the case of HCMV, IFNs are not used as antiviral drugs. Very early on, it became apparent that ‘human cytomegalovirus is relatively insensitive to the antiviral action of interferon in vitro, and that the rate of excretion of cytomegalovirus in the urine of a chronic human carrier was unaltered by circulating levels of interferon in the serum’ [47], suggesting that HCMV possesses potent IFN antagonists.

## 3. Posttranslational Modification of Proteins with Ubiquitin (Ubiquitination)

**Figure 1:**
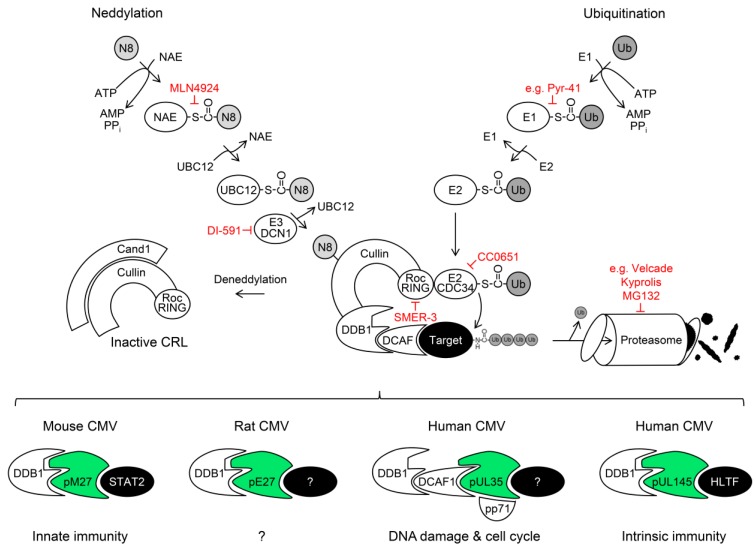
Exploitation of cellular cullin (Cul) RING ubiquitin (Ub) ligases (CRLs) by cytomegaloviruses. Simplified model of neddylation (left part), ubiquitination (right part), CRLs (central parts), and their exploitation by cytomegaloviral proteins (lower part). Drugs inhibiting the ubiquitin-proteasome system (UPS) or CRLs are highlighted in red. Please see the text for details and abbreviations.

Cells regulate the activity and stability of various proteins by posttranslational modification. One of the most important modifications is the conjugation with the 76 amino acid and approximately 8 kDa protein ubiquitin (Ub) [48] (see Figure 1 for a schematic overview). Ubiquitination (also referred to as ubiquitylation) usually occurs via a linkage between the C-terminal carboxyl group (COOH) of the Ub protein with side chains of the target protein. The most prevalent types of Ub linkage occur between the COOH group of the C-terminal glycine of Ub and a thiol group (SH) of a cysteine residue, or the formation of an isopeptide bond between the COOH group of Ub and an ε-amino group (NH_2_) of a lysine residue present in the target protein. Less frequently, other amino acids (e.g., serine, threonine, and tyrosine) or the N-terminus are modified by Ub conjugation [49]. Ubiquitination is a concerted and stepwise process, catalyzed by the consecutive activities of a Ub-activating enzyme (‘E1′), a Ub-conjugating enzyme (‘E2′), and a Ub ligase (‘E3′). 

The E1 enzyme activates Ub by ATP hydrolysis. Subsequently, the activated Ub is transferred from the E1 to the E2. From the E2, the Ub is linked to the target protein, usually catalyzed by a substrate-recognizing E3 ligase. The human genome encodes only a handful of enzymes with E1 activity [50,51], around 40 E2 enzymes, and several hundred E3 ligases [52]. The E1 as well the E2 enzymes and certain E3 ligases form thiol-linkages with Ub (structure: Ub-Gly-CO-S-Cys-enzyme), whereas Ub usually forms a covalent isopeptide bond with the target protein (structure: Ub-Gly-CO-NH-Lys-target). The situation is complicated by the fact that Ub harbors seven lysine residues (K6, K11, K27, K29, K33, K48, and K63) and the N-terminus (M1) [53], which can also be modified by subsequent ubiquitination events, resulting in a complex array of linear and branched, homotypic as well as heterotypic poly-Ub chains attached to different sites of various target proteins [54]. In addition to ubiquitination, Ub can also be subjected to other posttranslational modifications (e.g., phosphorylation, acetylation, sumoylation, neddylation, and ribosylation). Although the exact Ub code is far from being solved, it is clear that certain types of ubiquitination are mostly associated with defined down-stream events. For example, K48- and K11-linked poly-ubiquitinations usually cause the recognition by the proteasome and subsequent proteolytic degradation of the target protein, whereas K63-linked ubiquitination more often alters protein-protein interactions, resulting in a changed localization and/or activity of the modified protein without inducing degradation [55]. Since ubiquitination critically relies on very few E1 Ub activating enzymes, the pharmacologic inhibition of these E1s, using for example the drug PYR-41 [56], blocks Ub conjugation to a large extent.

## 4. The Proteasome

The 26S proteasome is a large multi-protein protease complex [57] usually composed of the 19S regulatory lid-like particle and the barrel-like 20S core particle. The core consists of two staked heptameric β-rings enclosed by two heptameric α-rings (structure: α_1-7_-(β_1-7_)_2_-α_1-7_) [58]. The subunits β_1_, β_2_, and β_5_ possess enzymatic protease activity. The lid covers the degradative chamber of the 20S core, recognizes proteins dedicated for proteolysis, stimulates the proteolytic activity of the core, exhibits ATPase and protein unfolding activity and deubiquitination activity. The subunits Rpn1, Rpn10, and Rpn13 recognize ubiquitinated proteins targeted for proteasomal degradation [59]. The target protein becomes partially unfolded and deubiquitinated in order to fit into the core, where the protein is cleaved into short peptides due to the chymotrypsin-like, trypsin-like, and peptidyl-glutamyl-peptide-hydrolyzing-like activity of the three catalytic β subunits. The proteasome can be inhibited by several different compounds like lactacystin [60], MG132 (also called carbobenzoxy-L-leucyl-L-leucyl-L-leucinal or Z-LLL-CHO [61]), and Velcade (also called PS-341 or Bortezomib [62]). In addition to these broad spectrum inhibitors of proteasomal degradation, subunit-specific inhibitors have been developed [63], such as PR-171 (also called Carfilzomib or Kyprolis) [64].

IFNs induce a marked change of the proteasome, called the formation of the immunoproteasome. By induction of the expression of three alternative subunits iβ1 (also called LMP2), iβ2 (also called MECL1), and iβ5 (also called LMP7), and simultaneous reduction of the abundance of the corresponding β subunits present in the constitutive proteasome, the peptidase specificity of the proteasome is altered [65]. Additionally, IFNγ induces the expression of the components of the 11S PA28 regulator particle. Recently, specific inhibitors of the immunoproteasome have been developed [66] like UK-101 [67] and ONX 0914 (previously known as PR-957) [68].

## 5. Cullin RING Ubiquitin Ligases (CRLs) and Their Regulation by Nedd8 Conjugation

Ub ligases are divided into three classes: the homologous to the E6-associated protein C-terminus (HECT), really interesting new gene (RING), and the RING-related U-box E3 ligases. RING and U-box E3 act as adaptor proteins, whereas HECT E3 ligases transiently accept and bind the Ub during the transfer to the target protein. The RING E3 ligases are further subdivided into two groups: enzymes, which act alone, and complex RING E3. Cullin (Cul) RING Ub ligases (CRL) are an important family belonging to the latter group of RING E3 enzymes. In CRLs, Roc1/2 (also called RocA/B or Rbx1/2) are the E2-binding RING Ub ligases. The best characterized CRL, the Skp1/Cullin 1/F-box (SCF) ligase complex, utilizes the E2 molecule UbcH5 for the initial ubiquitination, and Cdc34 for the subsequent poly-ubiquitination steps [69]. Roc1/2 is bridged to adapter proteins (e.g., DNA-damage binding protein 1 [DDB1]) and the associated substrate receptors (e.g., DDB1- and Cul4-associated factor 1 [DCAF1]) by cullin molecules (e.g., Cul4A/B). CRLs ubiquitinate numerous proteins and thus constitute important regulators of various biological processes including the DNA-damage response and the cell cycle. Based on their importance for the cell cycle regulation, CRLs received broad interest as targets for the development of anti-cancer drugs. The first-in-class inhibitor MLN4924 (also called Pevonedistat) takes advantage of a regulation mechanism, which is rather specific for CRLs. The cullin protein is conjugated with the Ub-like molecule Nedd8 in a process termed neddylation. CRLs devoid of Nedd8-conjugated cullins are functionally inactive, whereas CRLs containing neddylated cullins are enzymatically active due to the dissociation of the inhibitory molecule Cand1 [70]. Similar to the above-mentioned process of ubiquitination, neddylation is a three-step process executed by a specific set of E1, E2, and E3 enzymes. The Nedd8-activating enzyme (NAE) is the Nedd8-specific E1 enzyme. MLN4924 inhibits NAE activity [71]. Catalyzed by NAE, MLN4924 forms a covalent adduct with Nedd8 in the nucleotide-binding site of NAE, which resembles a Nedd8 adenylate but cannot be further utilized, thereby irreversibly blocking NAE activity. Since Nedd8 conjugation is required for efficient CRL activity, the inhibition of NAE by MLN4924 inactivates CRLs and stabilizes CRL target proteins. The activity of MLN4924 is rather specific, although Nedd8 also fulfils some functions beyond cullin modification [72].

Recently, two other CRL-inhibitory compounds have been described: (I) DI-591 inhibits the interaction of DCN1 with the Nedd8 E2 enzyme UBC12. This inhibits the neddylation and the activity of Cullin 3 [73]. (II) CRLs can also be inhibited by compounds, which target the corresponding E2 enzymes. This strategy is e.g., used by CC0651, which blocks Cdc34 [74].

### DDB1-Cullin 4A/B-RocA Complexes

As indicated by its name, DDB1 was initially identified as one of two components of the UV-DNA damage-binding complex, which is involved in nucleotide excision DNA repair [75,76]. Later it turned out that DDB1 is an important adapter protein for Cullin 4A (Cul4A) and Cullin 4B (Cul4B) [77]. DDB1 is a ~127 kDa protein adopting a four domain structure composed of a C-terminal domain and three seven-bladed β propeller domains [78]. DDB1 is an essential protein conserved from yeasts to humans. In mice, a null mutation results in embryonic lethality, while organ-specific deletions are associated with a loss of the respective tissue due to a p53-dependent elimination of cells that would otherwise proliferate [79,80]. In vitro, cells also die upon DDB1 ablation [81]. Via the recognition of WDxR and/or H-box motifs, DDB1 bridges several DDB1- and Cul4-associated factors (DCAFs) to Cul4A/B-Roc1 Ub ligases [82,83,84]. Through these DCAFs, target proteins are recruited and subjected to CRL-dependent ubiquitination and subsequent degradation by the proteasome, including the cell cycle regulators CTD1 and p21-CIP1 [85,86].

## 6. Exploitation of DDB1 and CRLs by Viruses

Several viruses encode accessory proteins, which cause the proteasomal degradation of host restriction factors by serving as adaptors bridging the target proteins to DDB1 and/or CRLs (reviewed in [87,88]). By this simple yet elegant mechanism, viruses exploit the cell intrinsic ubiquitin-proteasome system to defeat antiviral immunity. Well-known examples of viral exploitation of DDB1 and/or CRLs include the hepatitis B virus (HBV) protein HBx [84,89,90,91,92], the HIV-1- and HIV-2-encoded protein Vpr [93,94,95,96,97,98,99,100,101,102], HIV-2 Vpx [103,104,105,106,107,108,109], parainfluenza virus (PIV) V proteins [78,110,111,112], bovine herpesvirus 1 (BoHV-1) VP8 [113,114], murine gamma herpesvirus (MHV68) M2 [115], as well as the cytomegalovirus proteins pM27 [116,117,118], pUL35 [119], and pUL145 [120].

### 6.1. The DDB1-CRL-Interacting MCMV Protein pM27

The MCMV-encoded protein pM27 does not affect the MCMV replication in IFN-naïve fibroblasts in cell culture, but is essential for efficient replication in vivo [121,122,123]. The severe attenuation associated with the lack of *M27* results from the inability to counteract STAT2-dependent signaling. STAT2 is a transcription factor that mediates IFN type I and III signaling, and contributes to IFNγ signal transduction [44]. Accordingly, the STAT2-dependent aspect of IFNγ signaling is also inhibited by pM27 [40,44], as shown for the IFNγ-dependent induction of the immunoproteasome [124]. Although there is no indication that pM27 directly affects the IFNβ enhanceosome [125], certain cells like myeloid dendritic cells (mDCs), but not plasmacytoid dendritic cells (pDCs), exhibit increased IFN-I secretion upon infection with *M27*-deficient (ΔM27)-MCMV as compared to a wt-MCMV infection [126]. These seemingly contradictory findings can be reconciled when taking into account that in most cells—with the notable exception of pDCs—efficient IFN-I induction relies on an IFNAR1- and STAT2-dependent positive feedforward loop [127]. pM27 interferes with STAT2-dependent IFN signaling. Therefore, the most parsimonious explanation is that pM27 only affects IFN induction indirectly in cell types that utilize a STAT2-dependent positive feedforward loop for efficient IFN induction. Consistent with the IFN inhibitory function of pM27, ΔM27-MCMV exhibits an exaggerated IFN susceptibility in primary cells and several cell lines [40,44,116], and the phenotype of the *M27* deletion virus can be reverted by addition of pharmacologic inhibitors of IFN-Jak-STAT signaling (e.g., Ruxolitinib). Similarly, ΔM27-MCMV does not exhibit increased IFN susceptibility in STAT2-deficient cells in vitro. In mice, STAT2 deficiency largely, but not entirely, restores the ΔM27-MCMV replication, suggesting that STAT2 degradation is the most relevant, but not the sole function of pM27 [40]. In the presence of pM27, STAT2 is poly-ubiquitinated and subjected to proteasomal degradation [116]. pM27-dependent degradation of STAT2 does not require additional MCMV gene products (see e.g., [128]). Nevertheless, pM27 does not appear to possess E3 enzyme activity by itself. Instead, pM27 exploits a cellular CRL composed of DDB1, Cul4A or Cul4B, and supposedly Roc1 to ‘borrow’ E3 activity from the host [116,117]. Consistent with this molecular model, inhibition of Nedd8 conjugation by MLN4924 counteracts CRL activity [71] and restores STAT2 levels in the presence of pM27 [118]. 

The murid herpesviruses (MuHV) 8 and 2 encode the pM27 homologs pE27 and pR27, respectively. Interestingly, pE27 interacts with DDB1 and Cul4A/B, whereas pR27 only weakly interacts with DDB1 and Cul4B [117]. The HCMV-encoded homolog of pM27 is pUL27, which at best only weakly associates with DDB1 [117,129]. Additionally, pUL27 is dispensable for the HCMV-mediated STAT2 degradation [130]. However, MLN4924 also restores STAT2 in HCMV-infected cells [118], suggesting that HCMV encodes a yet to be identified protein that exploits CRLs to instruct the degradation of STAT2 in a manner analogous to pM27.

### 6.2. The DDB1-CRL-Interacting HCMV Protein pUL35 and its MCMV Homolog pM35

Similar to the phenotype of ΔM27-MCMV, a loss-of-function mutation of *M35* does not affect viral replication in cultured fibroblasts but attenuates the virus in mice [131]. Like pM27, the protein pM35 antagonizes innate immunity. However, in contrast to pM27, pM35 interferes directly with IFN induction by counteracting the NF-κB-dependent induction of IFNβ [132]. The HCMV homolog of the MCMV gene *M35* is *UL35*, which gives rise to two abundant protein isoforms of 22 kDa (p22-UL35; also called pUL35a) and 75 kDa (p75-UL35, often simply referred to as pUL35), respectively [133]. The protein p75-UL35 is a component of the virion. The deletion of the gene *UL35* severely affects HCMV replication in cell culture, especially at low multiplicities of infection [134], indicating notable differences between pUL35 and pM35. The two pUL35 isoforms physically interact with each other, with other viral proteins, and with several host proteins. The interaction of pUL35 and pp71-pUL82 is functionally relevant for modulating the activity of the viral major immediate early promoter (MIEP) [135]. The small p22-UL35 protein counteracts the MIEP-stimulating function of pp71, whereas p75-UL35 enhances it [133]. Additionally, pUL35 and pp71 were described to cooperate in inducing proteasomal degradation of BclAF1 early after HCMV infection [136]. Several other interactions of pUL35 with host proteins have been described: pUL35 interacts with PML, Sp100, and Daxx in PML bodies [137]. Another interaction of p75-UL35 with sorting nexin 5 (SNX5), affects the localization of glycoprotein B (gB) (also known as gpUL55) [138]. A proteomics study revealed that pUL35 also interacts with Usp7, DDB1, *DET1*- and *DDB1*-Associated *1* (DDA1), and DCAF1 to modulate the DNA repair response [119]. This suggests that pUL35 assembles an E3 ubiquitin ligase complex comprising DDB1, Cul4A, and Roc1.

### 6.3. The DDB1-CRL-Interacting HCMV Protein pUL145

The most recently described member of HCMV-encoded DDB1/CRL-interacting proteins is pUL145. In contrast to the adjacent genes *UL144* and *UL146*, *UL145* is highly conserved among different HCMV strains, suggesting its relevance for viral replication as well as the conservation of its interaction partner(s) [120,139,140,141]. The *UL145* gene gives rise to monocistronic as well as polycistronic transcripts [141,142]. Sequencing- and mass spectrometry-based methods detected *UL145* transcripts and the respective protein within the first six hours of infection [18,120].

The prediction of motifs for posttranslational modifications suggested a protein kinase C phosphorylation motif and one or two casein kinase II phosphorylation site(s) [139,141]. Additionally, Wang et al. proposed the existence of a zinc finger structure [140]. However, to our knowledge, a crystal structure is not yet available.

Recently, Nightingale et al. showed that pUL145 is responsible for a Cul4- and DDB1-dependent proteasomal degradation of the helicase-like transcription factor (HLTF), and additionally targets tumor protein p53-binding protein 1 (TP53BP1). HLTF has helicase and RING E3 ligase activity. Among other functions, HLTF promotes the lysine 63-linked poly-ubiquitination of the proliferating cell nuclear antigen (PCNA) [143,144], and displaces several proteins from stalled replication forks [145] to mediate error-free replication of damaged DNA [146]. Additionally, HLTF is reported to regulate transcription [147,148]. During HCMV infection, *HLTF* transcripts increase while HLTF protein levels decline. The HCMV-mediated degradation of HLTF was observed at four hours post infection. A *UL145* deletion virus lost the capacity to downregulate the HLTF protein levels. Furthermore, pUL145 co-precipitates components of the CRL complex comprising Cul4A, DDB1, and DDA1, indicating that pUL145 assembles a functional CRL complex to degrade the host restriction factor HLTF very early during HCMV infection [120]. Accordingly, HCMV-induced degradation of HLTF was found to be sensitive to MG132. These data highlight HLTF as an important host restriction factor that is counteracted by a viral antagonist to promote efficient virus replication. A role in viral restriction is also underscored by studies on HLTF degradation by the HIV-encoded protein Vpr (e.g., [98]). Interestingly, both HCMV and HIV achieve HLTF degradation by exploitation of cellular DDB1/CRL complexes.

## 7. The Ubiquitin-Proteasome System (UPS) as Antiviral Drug Target

Viruses encode proteins that are essential for replication (e.g., the viral polymerase). Additionally, viruses are obligate intracellular pathogens and critically rely on an array of host proteins for their replication. The latter are referred to as host dependency factors. For therapeutic interventions, three general strategies can be employed: (I) direct acting antivirals (DAA) target the function of essential viral enzymes; (II) Indirect acting antivirals (IAA) target host dependency factors that are essential for efficient viral replication. To limit the toxicity of the therapy, the targeted factors should be at least temporarily or locally dispensable for the well-being of the host; and (III) Immune-based strategies to eliminate the virus-infected host cell (e.g., by cytotoxic T lymphocytes). The advantage of DAAs is their selectivity for viruses and/or virus-infected cells. In principle, and notwithstanding potential off-target effects, DAAs have only very limited effects on uninfected bystander cells. However, due to their high mutation and replication rates, several viruses rapidly acquire resistance mutations rendering DAA therapies useless after a period of application. This is particularly prevalent in patient cohorts, which require continuous antiviral prophylaxis or treatment, such as AIDS patients or transplant recipients. In contrast, the barrier for resistance mutations to occur is far higher in the case of IAAs. However, this advantage comes at a price, since targeting host proteins is more likely to cause cytotoxicity. If the respective host protein was dispensable for the host, but essential for the replication of a relevant pathogen, the gene would likely be eliminated during evolution (since carriers of null-alleles would be resistant to the pathogen). Thus, the expression of a gene/protein serving as a host dependency factor for a relevant pathogen indicates its importance for the host. This is consistent with the finding that viral proteins preferentially target cellular hub and bottleneck proteins, which cannot be mutated or even eliminated by the host (discussed in [149]). Compounds that inhibit proteins involved in central pathways or structures of the cell (e.g., the ribosomes, the respiratory chain, or the UPS), are more likely to affect several viruses, resulting in increased therapeutic breadth—in exchange for elevated risk of toxicity. A large number of virus- and host-derived proteins are subjected to ubiquitination and proteasomal degradation. Accordingly, drugs inhibiting the UPS elicit potent antiviral activity in vitro against a variety of viruses—including CMVs [150,151,152]. This might sound like a dangerous approach, and, indeed, such drugs are rather toxic and have severe side-effects. However, the proteasome inhibitors Velcade (Bortezomib) and Kyprolis (Carfilzomib) have been approved by the FDA for the treatment of myelomas [153,154]. By applying treatment regimens with alternating phases of drug administration followed by drug-free recovery intervals, the exaggerated proteasome-dependency of tumor cells is exploited to outweigh detrimental effects on healthy cells. Similar approaches might be applicable for virus infections.

Unfortunately, there is a caveat to the application of UPS-targeting compounds against viruses: their immunosuppressive properties upon systemic administration. Velcade is a drug that inhibits cell proliferation. However, proliferation is essential for various aspects of effective immune responses. Additionally, the NF-κB pathway, which influences numerous aspects of immunity, relies on ubiquitination and subsequent proteasomal degradation of inhibitory proteins like IκBα. Hence, UPS inhibitors effectively block NF-κB signaling. Accordingly, Velcade administration renders mice more susceptible to lymphocytic choriomeningitis (mammarena-) virus (LCMV) [155]. In the case of the mouse-hepatitis coronavirus, the direct antiviral activity of Velcade observed in vitro is reversed to an exaggerated virus replication in vivo due to immunosuppression [156]. Similarly, humans undergo an increased frequency of herpesvirus reactivation events during Velcade therapy, as shown for the Varicella zoster virus [157] and HCMV [158]. Solutions to this issue might be the local/topical application of UPS inhibitors, targeted delivery, or the use of more specific drugs, which target only defined aspects of the UPS system and without globally inactivating ubiquitination or proteasomal proteolysis.

### CRLs as Antiviral Drug Targets

In our opinion, MLN4924 is a promising candidate for a drug eliciting antiviral activity by targeting a defined aspect of the UPS. By inhibiting NAE, MLN4924 blocks the Nedd8-dependent CRL activity [71]. MLN4924 has profound anti-tumor activity, e.g., against acute myeloid leukemia (AML) in cell culture models, primary patient specimens, and in animal xenograft models [159]. Based on these successes, several phase I and phase II clinical trials—using MLN4924 alone or in combination with other anti-tumor drugs—are running and, according to ClinicalTrials.gov, a phase III study is currently recruiting participants (see https://clinicaltrials.gov/ct2/results?cond=&term=mln4924&cntry=&state=&city=&dist=). The toxicity of MLN4924 has been thoroughly investigated in vitro and in animals. The most common side-effects observed in a phase I trial were fatigue and nausea, and ≥15% of patients reported adverse events, however, grade 4 adverse events and treatment-related deaths did not occur [160].

In cell culture, MLN4924 elicits potent antiviral activity against several clinically relevant viruses, including HIV [161], Rift Valley fever virus [162], influenza A virus [118,163], Kaposi sarcoma-associated herpesvirus (KSHV) [164,165], adenovirus [118], herpes simplex virus (HSV)-1 [118], HSV-2 [118], HBV [166], and HCMV [118]. The antiviral effect against HSV-1 was even evident against a multi-drug resistant isolate [118]. In the case of HCMV, highly significant inhibition of viral genome replication was observed at nanomolar concentrations. A comparison with GCV showed that MLN4924 significantly outperformed GCV at each tested concentration.

## 8. Conclusions and Outlook

There is compelling evidence that clinically relevant viruses (e.g., HIV, HBV, and HCMV) exploit the UPS and CRLs to shape their own proteome and to manipulate the host proteome to their own advantage. Several aspects of the UPS and CRLs are druggable [167,168]. However, the UPS and CRLs regulate the abundance of numerous proteins. Depending on the assessed cell type, the methodology, and the criteria employed, up to 19,000 sites in 5000 proteins are ubiquitinated [83], and the abundance of >80% of all proteins increases upon blockade of the proteasome [169]. Consistent with this global impact, the inhibition of the UPS is associated with substantial side-effects and toxicity. By increasing the specificity, less toxic regimens could be established. More subtle strategies might be to target neddylation, CRL-specific E2 enzymes, individual CRL complexes, or even substrate receptors of defined CRLs. CRLs are the primary but not the only target of neddylation [170,171,172], and global approaches have identified 496 Nedd8-accepting and Nedd8-associated proteins [170]. The combination of two experimental strategies identified 108 high confidence CRL substrates [85]. Thus, the drug MLN4924 inhibits only a fraction of all UPS-regulated proteins, but is still not entirely CRL-specific. E2- and CRL-specific approaches employing small molecules like CC0651 and SMER-3 [173] or DI-591, respectively, might further decrease toxicity. It will be interesting to test if such compounds possess antiviral activity against HCMV while being less toxic for the host.

**Figure 2:**
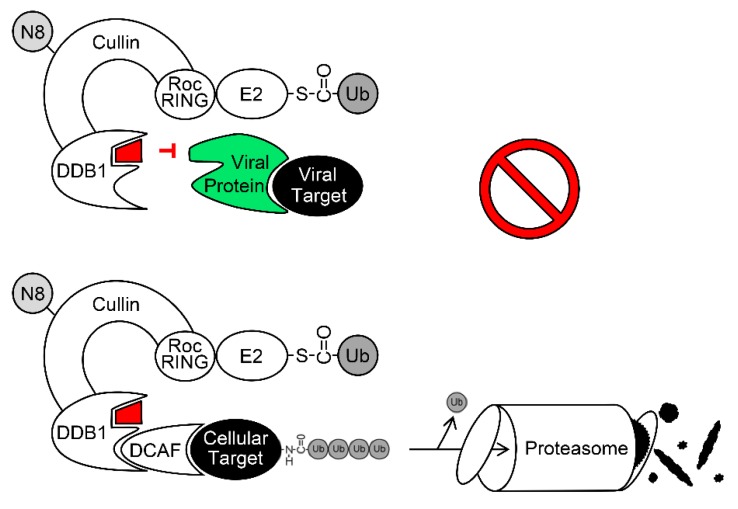
A strategy to target viral exploitation of CRLs without interfering with their cellular function. Please read the text for details.

An appealing future strategy might be to target CRLs and combine DAA and IAA aspects. Compounds blocking the association of viral proteins with CRLs without interfering with their genuine cellular function might represent a novel class of antiviral drugs (see Figure 2). If the antiviral activity is defined by a binding interface present in a cellular protein (e.g., the CRL), viral resistance should be rather difficult to achieve. If the cellular functions of the CRL are largely retained, toxicity should also be limited. For HBV, Sekiba et al. recently described a compound, which displaces HBx from DDB1 [174]. Using structural data already available for some of the complexes composed of cellular CRLs and viral proteins (see e.g., [78,84,175,176]), inhibitory compounds might be identified for other viruses. Thus, the molecular in-depth understanding of viral CRL-exploiting proteins and CRL interfaces could be instrumental for the design and development of novel antiviral compounds. Therefore, a mechanistic analysis of viral protein functions as well as cellular signaling and interaction networks will not only advance basic research but may also pave the way to new clinical applications.

## Abbreviations

AML	acute myeloid leukemia
BoHV-1	bovine herpesvirus 1
CRL	cullin RING ubiquitin ligases
CUL	cullin
Cul4A	cullin 4A
Cul4B	cullin 4B
DAA	direct acting antiviral
DCAF1	DDB1- and CUL4-associated factor 1
DDA1	DET1- and DDB1-Associated 1
DDB1	DNA-damage binding protein 1
E1	Ub-activating enzyme
E2	Ub-conjugating enzyme
E3	Ub ligase
GCV	Ganciclovir
GvHD	graft-versus-host disease
HAART	highly active antiretroviral therapy
HBV	hepatitis B virus
HCMV	human cytomegalovirus
HCV	hepatitis C virus
HECT	homologous to the E6-associated protein C-terminus
HHV-5	human herpesvirus 5
HIV	human immunodeficiency virus
HLTF	helicase-like transcription factor
IAA	indirect acting antiviral
IFN	interferon
IRepG	IFN-repressed gene
IRF	IFN-regulatory factor
ISG	IFN-stimulated gene
ISGF3	IFN-stimulated gene factor 3
Jak	janus kinase
MCMV	mouse cytomegalovirus
mDC	myeloid dendritic cell
MHV68	murine gamma herpesvirus 68
MIEP	major immediate early promoter
NAE	Nedd8-activating enzyme
ORF	open reading frame
PCNA	proliferating cell nuclear antigen
pDC	plasmacytoid dendritic cell
PIV	parainfluenza virus
RING	really interesting new gene
RL	repeat long
RS	repeat short
SCF	Skp1/cullin 1/F-box
STAT	signal transducer and activator of transcription
TP53BP1	tumor protein p53-binding protein 1
Tyk2	tyrosine kinase 2
Ub	ubiquitin
UL	unique long
UPS	ubiquitin-proteasome system
US	unique short
